# Supplementary material for: The Systems Biology Research Tool: evolvable open-source software
Source: BMC Syst Biol. 2008 Jun 29;2:55. doi: 10.1186/1752-0509-2-55 (PMC2446383; doi:10.1186/1752-0509-2-55)
Supplement: Additional file 1 — SBRT Archive. An archive of the current version of the Systems Biology Research Tool. [file 1752-0509-2-55-S1.zip › sbrt-1.4.0/doc/developers_guide/api/sbrt/shell/mng/class-use/ManagerInput.html]

Uses of Interface sbrt.shell.mng.ManagerInput


|  |  |  |
| --- | --- | --- |
| **Overview** \| **Package** \| **Class** \| **Use** \| **Tree** \| **Deprecated** \| **Index** \| **Help** | |  |
| PREV   NEXT | **FRAMES**    **NO FRAMES**     **All Classes** |  |


---


## **Uses of Interface sbrt.shell.mng.ManagerInput**

| Packages that use ManagerInput | |
| --- | --- |
| **sbrt** | Provides the class used to run the Systems Biology Research Tool. |
| **sbrt.shell** | Provides classes used by shell-level software. |
| **sbrt.shell.io** | Provides classes and interfaces for reading and writing files. |
| **sbrt.shell.mng** | Provides classes and interfaces for creating process managers. |
| **sbrt.shell.mng.algebra** | Provides classes for creating algebra process managers. |
| **sbrt.shell.mng.comb** | Provides classes for creating combinatorics process managers. |
| **sbrt.shell.mng.fba** | Provides classes and interfaces for creating Flux Balance Analysis process managers. |
| **sbrt.shell.mng.geom** | Provides classes and for creating geometry process managers. |
| **sbrt.shell.mng.graph\_theory** | Provides classes and for creating graph theoretical process managers. |
| **sbrt.shell.mng.stat** | Provides classes and interfaces for creating statistical process managers. |
| **sbrt.shell.mng.utilities** | Provides classes and interfaces for creating mathematical process managers. |

Uses of ManagerInput in sbrt

| Methods in sbrt with parameters of type ManagerInput | |
| --- | --- |
| `void` | `ProcessRunner.run(ManagerInput<java.lang.String,java.lang.String> processInput, java.io.OutputStream out)`             Executes the process specified in the provided process manager input. |
| `void` | `ProcessRunner.run(ManagerInput<java.lang.String,java.lang.String> processInput, java.io.PrintStream out)`             Executes the process specified in the provided process manager input. |
| `void` | `ProcessRunner.run(ManagerInput<java.lang.String,java.lang.String> processInput, java.io.PrintWriter out)`             Executes the process specified in the provided process manager input. |

Uses of ManagerInput in sbrt.shell

| Methods in sbrt.shell with parameters of type ManagerInput | |
| --- | --- |
| `static ProcessManager<java.lang.String,java.lang.String>` | `ProcessManagerFactory.make(ManagerInput<java.lang.String,java.lang.String> input, java.io.PrintWriter out)`             Prints a formatted string representation of the provided input to the provided print writer and returns its corresponding process manager. |
| `static <R,C> ProgramSolver<R,C>` | `ProgramSolverFactory.make(ManagerInput<java.lang.String,java.lang.String> input, java.io.PrintWriter out)`             Uses the information contained in the provided input to create a new program solver object. |
| `static void` | `ProcessManagerFactory.printInput(ManagerInput<java.lang.String,java.lang.String> input, java.io.PrintWriter out)`             Creates a formatted string representation of the provided input and prints it to the provided print writer. |

Uses of ManagerInput in sbrt.shell.io

| Classes in sbrt.shell.io that implement ManagerInput | |
| --- | --- |
| `class` | `ProcessInputFile`             This class is used to represent process input files. |

Uses of ManagerInput in sbrt.shell.mng

| Classes in sbrt.shell.mng that implement ManagerInput | |
| --- | --- |
| `class` | `MapManagerInput<K,V>`             This class is a concrete implemenation of `ManagerInput`. |

| Methods in sbrt.shell.mng with parameters of type ManagerInput | |
| --- | --- |
| `void` | `ProcessManager.check(ManagerInput<K,V> input)`             Throws an `ApplicationException` if this `ProcessManager` cannot understand the provided input. |
| `void` | `AbstractProcessManager.check(ManagerInput<java.lang.String,java.lang.String> input)`             Throws an ApplicationException if this `ProcessManager` cannot understand the provided input. |
| `static FileFormat` | `AbstractProcessManager.getFileFormat(ManagerInput<java.lang.String,java.lang.String> input)`             Returns the parsed value for `AbstractProcessManager.FILE_FORMAT` in the provided input. |
| `static TextInputFile<java.lang.String>` | `AbstractProcessManager.getFileNameSource(ManagerInput<java.lang.String,java.lang.String> input, java.io.PrintWriter out)`             Returns the parsed value for `AbstractProcessManager.OUTPUT_FILE_NAME_FILE` in the provided input and prints a descriptive message to the provided print writer. |
| `static int` | `AbstractProcessManager.getIterations(ManagerInput<java.lang.String,java.lang.String> input, java.io.PrintWriter out)`             Returns the parsed value for `AbstractProcessManager.ITERATIONS` in the provided input and prints a descriptive message to the provided print writer. |
| `static long` | `AbstractProcessManager.getSeed(ManagerInput<java.lang.String,java.lang.String> input, java.io.PrintWriter out)`             Returns the parsed value for `AbstractProcessManager.SEED` in the provided input and prints a descriptive message to the provided print writer. |
| `static FileFormat` | `AbstractProcessManager.getTextFileFormat(ManagerInput<java.lang.String,java.lang.String> input)`             Returns the parsed value for `AbstractProcessManager.FILE_FORMAT` in the provided input. |
| `static double` | `AbstractProcessManager.getZeroCutoff(ManagerInput<java.lang.String,java.lang.String> input)`             Returns the parsed value for `AbstractProcessManager.ZERO_CUTOFF` in the provided input. |
| `static double` | `AbstractProcessManager.getZeroCutoff(ManagerInput<java.lang.String,java.lang.String> input, java.io.PrintWriter out)`             Returns the parsed value for `AbstractProcessManager.ZERO_CUTOFF` in the provided input. |
| `void` | `ProcessManager.setInput(ManagerInput<K,V> input)`             Uses the provided input to prepare this process manager for execution. |
| `void` | `ProcessManager.setInput(ManagerInput<K,V> input, java.io.PrintWriter out)`             Uses the provided input to prepare this process manager for execution. |
| `void` | `AbstractProcessManager.setInput(ManagerInput<java.lang.String,java.lang.String> input)`             Uses the provided input to prepare this process manager for execution. |

Uses of ManagerInput in sbrt.shell.mng.algebra

| Methods in sbrt.shell.mng.algebra with parameters of type ManagerInput | |
| --- | --- |
| `static java.util.Map<LinearComb<java.lang.String>,java.lang.Double>` | `LinearSolverManager.getEquations(ManagerInput<java.lang.String,java.lang.String> input, java.io.PrintWriter out)`             Returns the set of linear equations contained in the file with the provided name. |
| `static MathematicaSolver<java.lang.String>` | `LinearSolverManager.getSolver(ManagerInput<java.lang.String,java.lang.String> input, java.io.PrintWriter out)`             Returns the linear system solver specified by `LinearSolverManager.KERNEL_LINK_CMD` in the provided input. |
| `void` | `LinearSolverManager.setInput(ManagerInput<java.lang.String,java.lang.String> input, java.io.PrintWriter out)`             Uses the provided input to prepare this process manager for execution. |
| `void` | `MultipleVectorsConverterManager.setInput(ManagerInput<java.lang.String,java.lang.String> input, java.io.PrintWriter out)`             Uses the provided input to prepare this process manager for execution. |
| `void` | `MatrixConverterManager.setInput(ManagerInput<java.lang.String,java.lang.String> input, java.io.PrintWriter out)`             Uses the provided input to prepare this process manager for execution. |
| `void` | `LinearCombConverterManager.setInput(ManagerInput<java.lang.String,java.lang.String> input, java.io.PrintWriter out)`             Uses the provided input to prepare this process manager for execution. |
| `void` | `SingleVectorConverterManager.setInput(ManagerInput<java.lang.String,java.lang.String> input, java.io.PrintWriter out)`             Uses the provided input to prepare this process manager for execution. |

Uses of ManagerInput in sbrt.shell.mng.comb

| Methods in sbrt.shell.mng.comb with parameters of type ManagerInput | |
| --- | --- |
| `static OutputFile<java.util.Set<java.lang.String>>` | `SingleElementUnionManager.getOutputFile(ManagerInput<java.lang.String,java.lang.String> input, java.io.PrintWriter out)`             Returns an output file with the name provided in the manager input. |
| `static java.util.List<java.util.Set<java.lang.String>>` | `SingleElementUnionManager.getSets(ManagerInput<java.lang.String,java.lang.String> input, java.io.PrintWriter out)`             Returns the list of sets contained in the file with the name contained in the provided input. |
| `void` | `StrictSingleElementUnionManager.setInput(ManagerInput<java.lang.String,java.lang.String> input, java.io.PrintWriter out)`             Uses the provided input to prepare this process manager for execution. |
| `void` | `SingleElementUnionManager.setInput(ManagerInput<java.lang.String,java.lang.String> input, java.io.PrintWriter out)`             Uses the provided input to prepare this process manager for execution. |

Uses of ManagerInput in sbrt.shell.mng.fba

| Methods in sbrt.shell.mng.fba with parameters of type ManagerInput | |
| --- | --- |
| `static Interval` | `RandomConstraintsManager.getActive(ManagerInput<java.lang.String,java.lang.String> input)`             Parses and returns the value for `RandomConstraintsManager.ACTIVE` in the provided manager input. |
| `static OutputFile<java.lang.Double>` | `SvInitialPointManager.getCentralityFile(ManagerInput<java.lang.String,java.lang.String> input, java.io.PrintWriter out)`             Parses and returns the value for `SvInitialPointManager.AVG_P_FILE_NAME` in the provided manager input. |
| `static CdHarFluxVectorComputer` | `CdHarFluxVectorManager.getComputer(ManagerInput<java.lang.String,java.lang.String> input, java.io.PrintWriter out)`             Creates and returns a coordinate-direction hit-and-run flux vector computer using the provided input. |
| `static ExtremeCurrentComputer` | `ExtremeCurrentManager.getComputer(ManagerInput<java.lang.String,java.lang.String> input, java.io.PrintWriter out)`             Returns an extreme current computer created from the provided input. |
| `static ReverseRxnFluxIntervalComputer` | `ReverseRxnFluxIntervalManager.getComputer(ManagerInput<java.lang.String,java.lang.String> input, java.io.PrintWriter out)`             Returns the flux interval computer specified by the provided input. |
| `static SimpleFluxIntervalComputer` | `SimpleFluxIntervalManager.getComputer(ManagerInput<java.lang.String,java.lang.String> input, java.io.PrintWriter out)`             Returns the flux interval computer specified by the provided input. |
| `static FbaOptComputer` | `FbaOptManager.getComputer(ManagerInput<java.lang.String,java.lang.String> input, java.io.PrintWriter out)`             Creates and returns an FBA optimization computer based on the provided input. |
| `static MsFluxIntervalComputer` | `MsFluxIntervalManager.getComputer(ManagerInput<java.lang.String,java.lang.String> input, java.io.PrintWriter out)`             Returns the flux interval computer specified by the provided input. |
| `static FbaOptComputer` | `InitialPointManager.getComputer(ManagerInput<java.lang.String,java.lang.String> input, java.io.PrintWriter out)`             Creates and returns an FBA optimization computer based on the provided input. |
| `static CdHarFluxVectorComputer` | `CdHarFluxVectorManager.getComputer(ManagerInput<java.lang.String,java.lang.String> input, java.io.PrintWriter out, IrrevRxnFluxome fluxome, double tolerance)`             Creates and returns a coordinate-direction hit-and-run flux vector computer using the provided input. |
| `static FluxConstraints` | `FbaOptManager.getConstraints(ManagerInput<java.lang.String,java.lang.String> input, java.io.PrintWriter out, Fluxome fluxome)`             Opens the file denoted by `FbaOptManager.CONSTRAINTS_FILE` in the provided manager input and returns the set of flux constraints it contains. |
| `static FluxConstraints` | `RandomFluxVectorManager.getConstraints(ManagerInput<java.lang.String,java.lang.String> input, java.io.PrintWriter out, Fluxome fluxome)`             Opens the file denoted by `FbaOptManager.CONSTRAINTS_FILE` in the provided manager input and returns the set of flux constraints it contains. |
| `static double` | `FbaManager.getConstraintTolerance(ManagerInput<java.lang.String,java.lang.String> input)`             Parses and returns the value for `FbaManager.CONSTRAINT_TOLERANCE` in the provided manager input. |
| `static double` | `FbaManager.getConstraintTolerance(ManagerInput<java.lang.String,java.lang.String> input, java.io.PrintWriter out)`             Parses and returns the value for `FbaManager.CONSTRAINT_TOLERANCE` in the provided manager input. |
| `static MapOutputFile<java.lang.String,java.lang.Double>` | `InitialPointManager.getCVectorFile(ManagerInput<java.lang.String,java.lang.String> input, java.io.PrintWriter out, Fluxome fluxome)`             Returns an empty output file that is suitable for storing a single C vector with the name denoted by `InitialPointManager.P_VECTOR_FILE_NAME` in the provided input. |
| `static FluxIntervalInputFile` | `IntervalVariationManager.getCvInputFile(ManagerInput<java.lang.String,java.lang.String> input, java.io.PrintWriter out, Fluxome fluxome)`             Returns the constraint variation input with the name denoted by `IntervalVariationManager.CV_FILE` in the provided manager input. |
| `static java.util.List<LinearComb<java.lang.String>>` | `FluxCapManager.getCycles(ManagerInput<java.lang.String,java.lang.String> input, java.io.PrintWriter out, Fluxome fluxome)`             Opens the file denoted by `FluxCapManager.CYCLE_FILE` in the provided manager input and returns the list of cycles it contains. |
| `static java.util.Set<java.lang.String>` | `FbaOptManager.getDataHeaders(ManagerInput<java.lang.String,java.lang.String> input, Fluxome fluxome)`             Parses and returns the value for `AbstractProcessManager.DATA_HEADERS` in the provided manager input. |
| `static RxnDeletionComputer` | `RxnDeletionManager.getDeletionComputer(ManagerInput<java.lang.String,java.lang.String> input, java.io.PrintWriter out)`             Returns a reaction deletion computer created from the provided input. |
| `static InputFile<java.util.Set<java.lang.String>>` | `CatalystDeletionManager.getDeletiontFile(ManagerInput<java.lang.String,java.lang.String> input, java.io.PrintWriter out, CatalyzedFluxome fluxome)`             Parses and returns the value for `CatalystDeletionManager.CATALYST_DELETION_FILE` in the provided manager input. |
| `static UnmodifiableMap<java.lang.String,java.util.Set<LinearComb<java.lang.String>>>` | `MsFluxIntervalManager.getFluxCaps(ManagerInput<java.lang.String,java.lang.String> input, java.io.PrintWriter out, Fluxome fluxome)`             Returns the flux caps contained in the file with the name specified by `MsFluxIntervalManager.FLUX_CAP_FILE` in the provided input. |
| `static MapInputFile<java.lang.String,Interval>` | `FluxPlasticityManager.getFluxIntervalFile(ManagerInput<java.lang.String,java.lang.String> input, java.io.PrintWriter out, Fluxome fluxome)`             Returns the flux interval input with the name denoted by `FluxPlasticityManager.FLUX_INTERVAL_FILE` in the provided manager input. |
| `static IrrevRxnFluxome` | `FbaOptManager.getFluxome(ManagerInput<java.lang.String,java.lang.String> input, java.io.PrintWriter out)`             Creates and returns a fluxome based on the provided input. |
| `static IrrevRxnFluxome` | `FbaManager.getFluxome(ManagerInput<java.lang.String,java.lang.String> input, java.io.PrintWriter out)`             Opens the file denoted by `FbaManager.RXN_FILE` in the provided manager input and returns the fluxome it contains. |
| `static IrrevRxnFluxome` | `RandomFluxVectorManager.getFluxome(ManagerInput<java.lang.String,java.lang.String> input, java.io.PrintWriter out)`             Creates and returns a fluxome based on the provided input. |
| `static IrrevRxnFluxome` | `FbaOptManager.getFluxome(ManagerInput<java.lang.String,java.lang.String> input, java.io.PrintWriter out, double tolerance)`             Creates and returns a fluxome based on the provided input. |
| `static IrrevRxnFluxome` | `InitialPointManager.getFluxome(ManagerInput<java.lang.String,java.lang.String> input, java.io.PrintWriter out, double tolerance)`             Creates and returns a fluxome based on the provided input. |
| `static LinearSystemSolution<java.lang.String>` | `FbaManager.getFluxomeSolution(ManagerInput<java.lang.String,java.lang.String> input, java.io.PrintWriter out, Fluxome fluxome)`             Opens the file denoted by `FbaManager.SYSTEM_SOLUTION_FILE` in the provided manager input and returns the linear system solution it contains. |
| `static java.util.Map<java.lang.String,java.lang.Double>` | `CdHarFluxVectorManager.getFluxVector(ManagerInput<java.lang.String,java.lang.String> input, java.io.PrintWriter out, Fluxome fluxome, double tolerance)`             Opens the file denoted by `CdHarManager.INITIAL_POINT_FILE` in the provided manager input and returns the flux vector it contains. |
| `static java.util.Map<java.lang.String,java.lang.Double>` | `RandomFluxVectorManager.getFluxVector(ManagerInput<java.lang.String,java.lang.String> input, java.io.PrintWriter out, Fluxome fluxome, double tolerance)`             Opens the file denoted by `FbaManager.FLUX_VECTOR_FILE` in the provided manager input and returns the flux vector it contains. |
| `static MapOutputFile<java.lang.String,java.lang.Double>` | `SvInitialPointManager.getFluxVectorFile(ManagerInput<java.lang.String,java.lang.String> input, java.io.PrintWriter out, Fluxome fluxome)`             Returns an empty output file that is suitable for storing multiple flux vectors. |
| `static MapOutputFile<java.lang.String,java.lang.Double>` | `InitialPointManager.getFluxVectorFile(ManagerInput<java.lang.String,java.lang.String> input, java.io.PrintWriter out, Fluxome fluxome)`             Returns an empty output file that is suitable for storing a single flux vector. |
| `static MapInputFile<java.lang.String,java.lang.Double>` | `FluxActivityManager.getFluxVectorFile(ManagerInput<java.lang.String,java.lang.String> input, java.io.PrintWriter out, Fluxome fluxome)`             Returns the flux vector input file specified by the provided input. |
| `static Interval` | `RandomConstraintsManager.getInactive(ManagerInput<java.lang.String,java.lang.String> input)`             Parses and returns the value for `RandomConstraintsManager.INACTIVE` in the provided manager input. |
| `static InputFile<java.util.Map<java.lang.String,java.lang.Double>>` | `SvCdHarFluxVectorManager.getInitialFluxVectorFile(ManagerInput<java.lang.String,java.lang.String> input, java.io.PrintWriter out, Fluxome fluxome)`             Returns the input file containing a collection of initial flux vectors. |
| `static InputFile<java.util.Set<java.lang.String>>` | `RxnDeletionManager.getInputFile(ManagerInput<java.lang.String,java.lang.String> input, java.io.PrintWriter out, Fluxome fluxome)`             Parses and returns the value for `RxnDeletionManager.RXN_DELETION_FILE` in the provided manager input. |
| `static TextInputFile<LinearComb<java.lang.String>>` | `ObjectiveFunctionManager.getInputFile(ManagerInput<java.lang.String,java.lang.String> input, java.io.PrintWriter out, Fluxome fluxome)`             Returns the file denoted by `ObjectiveFunctionManager.OBJ_FUNCT_FILE` in the provided manager input. |
| `static FluxConstraints` | `InitialPointManager.getIntervals(ManagerInput<java.lang.String,java.lang.String> input, java.io.PrintWriter out, Fluxome fluxome)`             Opens the file denoted by `InitialPointManager.FLUX_INTERVAL_FILE` in the provided manager input and returns the set of flux intervals it contains. |
| `static MultiOutputFiles<ComoFile<java.lang.String,java.lang.Double>,java.util.Map<? extends java.lang.String,? extends java.lang.Double>>` | `SvCdHarFluxVectorManager.getMulitComoFiles(ManagerInput<java.lang.String,java.lang.String> input, java.io.PrintWriter out, java.util.Iterator<java.lang.String> fileNameSource, Fluxome fluxome)`             Returns a source of output files. |
| `static MultiOutputFiles<ColumnOrFbaOptOutputFile,FbaOptComputer.Results>` | `FbaOptManager.getMultiColOrOutputFiles(ManagerInput<java.lang.String,java.lang.String> input, java.io.PrintWriter out, java.util.Iterator<java.lang.String> fileNameSource, java.lang.String fileNameSourceName, Fluxome fluxome)`             Please consult the source code for a complete description of this method's actions. |
| `static MultiOutputFiles<? extends FbaOptOutputFile,FbaOptComputer.Results>` | `FbaOptManager.getMultiFileOutput(ManagerInput<java.lang.String,java.lang.String> input, java.io.PrintWriter out, java.util.Iterator<java.lang.String> fileNameSource, java.lang.String fileNameSourceName, Fluxome fluxome)`             Please consult the source code for a complete description of this method's actions. |
| `static MapOutputFile<java.lang.String,java.lang.Double>` | `CdHarFluxVectorManager.getMultiFluxVectorOutputFile(ManagerInput<java.lang.String,java.lang.String> input, java.io.PrintWriter out, Fluxome fluxome)`             Returns an empty output file that is suitable for storing multiple flux vectors. |
| `static MapOutputFile<java.lang.String,java.lang.Double>` | `FbaManager.getMultiFluxVectorOutputFile(ManagerInput<java.lang.String,java.lang.String> input, java.io.PrintWriter out, Fluxome fluxome)`             Returns an empty output file that is suitable for storing multiple flux vectors. |
| `static ComoFile<java.lang.String,Interval>` | `SimpleFluxIntervalManager.getMultiResultsOutputFile(ManagerInput<java.lang.String,java.lang.String> input, java.io.PrintWriter out, Fluxome fluxome)`             Returns an empty output file that is suitable for storing multiple sets of flux interval results. |
| `static FbaOptOutputFile` | `FbaOptManager.getMultiResultsOutputFile(ManagerInput<java.lang.String,java.lang.String> input, java.io.PrintWriter out, Fluxome fluxome)`             Returns an empty output file that is suitable for storing multiple FBA optimization results. |
| `static MultiOutputFiles<RowOrFbaOptOutputFile,FbaOptComputer.Results>` | `FbaOptManager.getMultiRowOrOutputFiles(ManagerInput<java.lang.String,java.lang.String> input, java.io.PrintWriter out, java.util.Iterator<java.lang.String> fileNameSource, Fluxome fluxome)`             Please consult the source code for a complete description of this method's actions. |
| `static MultiOutputFiles<SerFbaOptOutputFile,FbaOptComputer.Results>` | `FbaOptManager.getMultiSerOutputFiles(ManagerInput<java.lang.String,java.lang.String> input, java.io.PrintWriter out, java.util.Iterator<java.lang.String> fileNameSource, java.lang.String fileNameSourceName, Fluxome fluxome)`             Please consult the source code for a complete description of this method's actions. |
| `static LinearComb<java.lang.String>` | `FbaOptManager.getObjectiveFunction(ManagerInput<java.lang.String,java.lang.String> input, java.io.PrintWriter out, Fluxome fluxome)`             Parses and returns the value for `FbaOptManager.OBJECTIVE_FUNCTION` in the provided manager input. |
| `static OutputFile<java.lang.Integer>` | `SvCdHarFluxVectorManager.getObsructedMovesFile(ManagerInput<java.lang.String,java.lang.String> input, java.io.PrintWriter out)`             Returns an output file used to store the number of obstructed moves per flux space. |
| `static int` | `RandomConstraintsManager.getOnFluxes(ManagerInput<java.lang.String,java.lang.String> input, java.io.PrintWriter out)`             Parses and returns the value for `RandomConstraintsManager.NUMBER_OF_ON_FLUXES` in the provided manager input. |
| `static OptimizationSense` | `FbaOptManager.getOptimizationSense(ManagerInput<java.lang.String,java.lang.String> input)`             Parses and returns the value for `FbaOptManager.OPTIMIZATION_SENSE` in the provided manager input. |
| `static ComoFile<java.lang.String,java.lang.Double>` | `ExtremeCurrentManager.getOutputFile(ManagerInput<java.lang.String,java.lang.String> input, java.io.PrintWriter out, Fluxome fluxome, java.util.Set<java.lang.String> rxnNames)`             Returns an output file to which extreme currents can be written. |
| `static MapTextOutputFile<java.lang.String,Interval>` | `RandomConstraintsManager.getOutputFile(ManagerInput<java.lang.String,java.lang.String> input, java.io.PrintWriter out, IrrevRxnFluxome fluxome)`             Returns an empty output file that is suitable for storing multiple sets of flux constraints. |
| `static MapTextOutputFile<java.lang.String,Interval>` | `RandomEnvManager.getOutputFile(ManagerInput<java.lang.String,java.lang.String> input, java.io.PrintWriter out, IrrevRxnFluxome fluxome)`             Returns an empty output file that is suitable for storing multiple environments. |
| `static TextOutputFile<LinearComb<java.lang.String>>` | `RandomObjectiveManager.getOutputFile(ManagerInput<java.lang.String,java.lang.String> input, java.io.PrintWriter out, IrrevRxnFluxome fluxome)`             Returns an empty output file that is suitable for storing randomly generated objective functions. |
| `static double` | `InitialPointManager.getPIntercept(ManagerInput<java.lang.String,java.lang.String> input, java.io.PrintWriter out)`             Returns the parsed value for `InitialPointManager.P_MAX` in the provided input. |
| `static java.util.Set<java.lang.String>` | `FbaManager.getRxnNameHeaders(ManagerInput<java.lang.String,java.lang.String> input, Fluxome fluxome)`             Parses and returns the value for `AbstractProcessManager.DATA_HEADERS` in the provided manager input. |
| `static int` | `FbaOptManager.getSafetyLevel(ManagerInput<java.lang.String,java.lang.String> input, java.io.PrintWriter out)`             Parses and returns the value for `FbaOptManager.SAFETY_LEVEL` in the provided manager input. |
| `static MapOutputFile<java.lang.String,java.lang.Double>` | `FbaManager.getSingleFluxVectorOutputFile(ManagerInput<java.lang.String,java.lang.String> input, java.io.PrintWriter out, Fluxome fluxome)`             Returns an empty output file that is suitable for storing a single flux vector. |
| `static RomoFile<java.lang.String,Interval>` | `SimpleFluxIntervalManager.getSingleResultOutputFile(ManagerInput<java.lang.String,java.lang.String> input, java.io.PrintWriter out, Fluxome fluxome)`             Returns an empty output file that is suitable for storing a single set of flux interval results. |
| `static FbaOptOutputFile` | `FbaOptManager.getSingleResultOutputFile(ManagerInput<java.lang.String,java.lang.String> input, java.io.PrintWriter out, Fluxome fluxome)`             Returns an empty output file that is suitable for storing a single FBA optimization result. |
| `static FluxIntervalInputFile` | `IntervalVariationManager.getSvInputFile(ManagerInput<java.lang.String,java.lang.String> input, java.io.PrintWriter out, Fluxome fluxome)`             Returns the space variation input with the name denoted by `IntervalVariationManager.FLUX_INTERVAL_FILE` in the provided manager input. |
| `void` | `WwFluxomeReducerManager.setInput(ManagerInput<java.lang.String,java.lang.String> input, java.io.PrintWriter out)`             Uses the provided input to prepare this process manager for execution. |
| `void` | `RandomConstraintsManager.setInput(ManagerInput<java.lang.String,java.lang.String> input, java.io.PrintWriter out)`             Uses the provided input to prepare this process manager for execution. |
| `void` | `CdHarFluxVectorManager.setInput(ManagerInput<java.lang.String,java.lang.String> input, java.io.PrintWriter out)`             Uses the provided input to prepare this process manager for execution. |
| `void` | `RandomEnvManager.setInput(ManagerInput<java.lang.String,java.lang.String> input, java.io.PrintWriter out)`             Uses the provided input to prepare this process manager for execution. |
| `void` | `ExtremeCurrentManager.setInput(ManagerInput<java.lang.String,java.lang.String> input, java.io.PrintWriter out)`             Uses the provided input to prepare this process manager for execution. |
| `void` | `CvFbaOptManager.setInput(ManagerInput<java.lang.String,java.lang.String> input, java.io.PrintWriter out)`             Uses the provided input to prepare this process manager for execution. |
| `void` | `BiggSbmlTranslaterManager.setInput(ManagerInput<java.lang.String,java.lang.String> input, java.io.PrintWriter out)`             Uses the provided input to prepare this process manager for execution. |
| `void` | `NetworkInfoManager.setInput(ManagerInput<java.lang.String,java.lang.String> input, java.io.PrintWriter out)`             Uses the provided input to prepare this process manager for execution. |
| `void` | `StoichVariationManager.setInput(ManagerInput<java.lang.String,java.lang.String> input, java.io.PrintWriter out)`             Uses the provided input to prepare this process manager for execution. |
| `void` | `RxnRemovalManager.setInput(ManagerInput<java.lang.String,java.lang.String> input, java.io.PrintWriter out)`             Uses the provided input to prepare this process manager for execution. |
| `void` | `FluxCapManager.setInput(ManagerInput<java.lang.String,java.lang.String> input, java.io.PrintWriter out)`             Uses the provided input to prepare this process manager for execution. |
| `void` | `ReverseRxnFluxIntervalManager.setInput(ManagerInput<java.lang.String,java.lang.String> input, java.io.PrintWriter out)`             Uses the provided input to prepare this process manager for execution. |
| `void` | `SimpleFluxIntervalManager.setInput(ManagerInput<java.lang.String,java.lang.String> input, java.io.PrintWriter out)`             Uses the provided input to prepare this process manager for execution. |
| `void` | `FbaOptManager.setInput(ManagerInput<java.lang.String,java.lang.String> input, java.io.PrintWriter out)`             Uses the provided input to prepare this process manager for execution. |
| `void` | `RxnDeletionManager.setInput(ManagerInput<java.lang.String,java.lang.String> input, java.io.PrintWriter out)`             Uses the provided input to prepare this process manager for execution. |
| `void` | `CvRxnDeletionManager.setInput(ManagerInput<java.lang.String,java.lang.String> input, java.io.PrintWriter out)`             Uses the provided input to prepare this process manager for execution. |
| `void` | `ObjectiveFunctionManager.setInput(ManagerInput<java.lang.String,java.lang.String> input, java.io.PrintWriter out)`             Uses the provided input to prepare this process manager for execution. |
| `void` | `SlpCycleIdManager.setInput(ManagerInput<java.lang.String,java.lang.String> input, java.io.PrintWriter out)`             Uses the provided input to prepare this process manager for execution. |
| `void` | `SimpleRxnFileTransManager.setInput(ManagerInput<java.lang.String,java.lang.String> input, java.io.PrintWriter out)`             Uses the provided input to prepare this process manager for execution. |
| `void` | `SvInitialPointManager.setInput(ManagerInput<java.lang.String,java.lang.String> input, java.io.PrintWriter out)`             Uses the provided input to prepare this process manager for execution. |
| `void` | `MetatoolFileWriterManager.setInput(ManagerInput<java.lang.String,java.lang.String> input, java.io.PrintWriter out)`             Uses the provided input to prepare this process manager for execution. |
| `void` | `SbmlWriterManager.setInput(ManagerInput<java.lang.String,java.lang.String> input, java.io.PrintWriter out)`             Uses the provided input to prepare this process manager for execution. |
| `void` | `CvCatalystDeletionManager.setInput(ManagerInput<java.lang.String,java.lang.String> input, java.io.PrintWriter out)`             Uses the provided input to prepare this process manager for execution. |
| `void` | `MsFluxomeReducerManager.setInput(ManagerInput<java.lang.String,java.lang.String> input, java.io.PrintWriter out)`             Uses the provided input to prepare this process manager for execution. |
| `void` | `FbaSolverManager.setInput(ManagerInput<java.lang.String,java.lang.String> input, java.io.PrintWriter out)`             Uses the provided input to prepare this process manager for execution. |
| `void` | `MmaNotebookManager.setInput(ManagerInput<java.lang.String,java.lang.String> input, java.io.PrintWriter out)`             Uses the provided input to prepare this process manager for execution. |
| `void` | `BiggSbmlReaderManager.setInput(ManagerInput<java.lang.String,java.lang.String> input, java.io.PrintWriter out)`             Uses the provided input to prepare this process manager for execution. |
| `void` | `CvRevRxnFluxIntervalManager.setInput(ManagerInput<java.lang.String,java.lang.String> input, java.io.PrintWriter out)`             Uses the provided input to prepare this process manager for execution. |
| `void` | `FluxPlasticityManager.setInput(ManagerInput<java.lang.String,java.lang.String> input, java.io.PrintWriter out)`             Uses the provided input to prepare this process manager for execution. |
| `void` | `MsFluxIntervalManager.setInput(ManagerInput<java.lang.String,java.lang.String> input, java.io.PrintWriter out)`             Uses the provided input to prepare this process manager for execution. |
| `void` | `RandomObjectiveManager.setInput(ManagerInput<java.lang.String,java.lang.String> input, java.io.PrintWriter out)`             Uses the provided input to prepare this process manager for execution. |
| `void` | `CvMsFluxIntervalManager.setInput(ManagerInput<java.lang.String,java.lang.String> input, java.io.PrintWriter out)`             Uses the provided input to prepare this process manager for execution. |
| `void` | `PalssonSbmlTranslaterManager.setInput(ManagerInput<java.lang.String,java.lang.String> input, java.io.PrintWriter out)`             Uses the provided input to prepare this process manager for execution. |
| `void` | `SvCdHarFluxVectorManager.setInput(ManagerInput<java.lang.String,java.lang.String> input, java.io.PrintWriter out)`             Uses the provided input to prepare this process manager for execution. |
| `void` | `CvSimpleFluxIntervalManager.setInput(ManagerInput<java.lang.String,java.lang.String> input, java.io.PrintWriter out)`             Uses the provided input to prepare this process manager for execution. |
| `void` | `CatalystDeletionManager.setInput(ManagerInput<java.lang.String,java.lang.String> input, java.io.PrintWriter out)`             Uses the provided input to prepare this process manager for execution. |
| `void` | `InitialPointManager.setInput(ManagerInput<java.lang.String,java.lang.String> input, java.io.PrintWriter out)`             Uses the provided input to prepare this process manager for execution. |
| `void` | `PalssonSbmlReaderManager.setInput(ManagerInput<java.lang.String,java.lang.String> input, java.io.PrintWriter out)`             Uses the provided input to prepare this process manager for execution. |
| `void` | `CvObjectiveFunctionManager.setInput(ManagerInput<java.lang.String,java.lang.String> input, java.io.PrintWriter out)`             Uses the provided input to prepare this process manager for execution. |
| `void` | `FluxActivityManager.setInput(ManagerInput<java.lang.String,java.lang.String> input, java.io.PrintWriter out)`             Uses the provided input to prepare this process manager for execution. |

Uses of ManagerInput in sbrt.shell.mng.geom

| Methods in sbrt.shell.mng.geom with parameters of type ManagerInput | |
| --- | --- |
| `static double` | `CdHarManager.getConstraintTolerance(ManagerInput<java.lang.String,java.lang.String> input, java.io.PrintWriter out)`             Parses and returns the value for `CdHarManager.CONSTRAINT_TOLERANCE` in the provided manager input. |
| `static java.util.Map<java.lang.String,java.lang.Double>` | `CdHarManager.getInitialPoint(ManagerInput<java.lang.String,java.lang.String> input, java.io.PrintWriter out, java.util.Set<java.lang.String> variables)`             Opens the file with the name denoted by `CdHarManager.INITIAL_POINT_FILE` and return the vector it contains. |
| `static int` | `CdHarManager.getMaxObstructedMoves(ManagerInput<java.lang.String,java.lang.String> input, java.io.PrintWriter out)`             Parses and returns the value for `CdHarManager.MAX_OBSTRUCTED_MOVES` in the provided manager input. |
| `static double` | `CdHarManager.getMinChordLength(ManagerInput<java.lang.String,java.lang.String> input, java.io.PrintWriter out)`             Parses and returns the value for `CdHarManager.MIN_CHORD_LENGTH` in the provided manager input. |
| `static ComoFile<java.lang.String,java.lang.Double>` | `CdHarManager.getOutputFile(ManagerInput<java.lang.String,java.lang.String> input, java.io.PrintWriter out, java.util.Set<java.lang.String> headers)`             Returns an output file with the name provided in the manager input. |
| `static java.util.Set<ParallelHyperplanes<java.lang.String>>` | `CdHarManager.getPlanes(ManagerInput<java.lang.String,java.lang.String> input, java.io.PrintWriter out)`             Returns the list of sets contained in the file with the name contained in the provided input. |
| `static int` | `CdHarManager.getPointsPerPoint(ManagerInput<java.lang.String,java.lang.String> input, java.io.PrintWriter out)`             Parses and returns the value for `CdHarManager.POINTS_PER_POINT` in the provided manager input. |
| `void` | `CdHarManager.setInput(ManagerInput<java.lang.String,java.lang.String> input, java.io.PrintWriter out)`             Uses the provided input to prepare this process manager for execution. |

Uses of ManagerInput in sbrt.shell.mng.graph\_theory

| Methods in sbrt.shell.mng.graph\_theory with parameters of type ManagerInput | |
| --- | --- |
| `static DirectedGraph<java.lang.String>` | `PathIdManager.getGraph(ManagerInput<java.lang.String,java.lang.String> input, java.io.PrintWriter out)`             Opens the file denoted by `PathIdManager.EDGE_FILE` in the provided manager input and returns the directed graph it contains. |
| `static TextOutputFile<java.util.List<java.lang.String>>` | `CycleIdManager.getOutputFile(ManagerInput<java.lang.String,java.lang.String> input)`             Creates a returns an output file if one is specified in the provided input. |
| `static TextOutputFile<java.util.List<java.lang.String>>` | `CycleIdManager.getOutputFile(ManagerInput<java.lang.String,java.lang.String> input, java.io.PrintWriter out)`             Creates a returns an output file if one is specified in the provided input. |
| `void` | `CycleIdManager.setInput(ManagerInput<java.lang.String,java.lang.String> input, java.io.PrintWriter out)`             Uses the provided input to prepare this process manager for execution. |
| `void` | `UniqueCycleIdManager.setInput(ManagerInput<java.lang.String,java.lang.String> input, java.io.PrintWriter out)`             Uses the provided input to prepare this process manager for execution. |
| `void` | `PathIdManager.setInput(ManagerInput<java.lang.String,java.lang.String> input, java.io.PrintWriter out)`             Uses the provided input to prepare this process manager for execution. |

Uses of ManagerInput in sbrt.shell.mng.stat

| Methods in sbrt.shell.mng.stat with parameters of type ManagerInput | |
| --- | --- |
| `static UnmodifiableMap<java.lang.String,java.lang.Double>` | `CorrelationManager.getXValues(ManagerInput<java.lang.String,java.lang.String> input, java.io.PrintWriter out)`             Returns the parsed value for `CorrelationManager.X_FILE_NAME` in the provided input and prints a descriptive message to the provided print writer. |
| `static UnmodifiableMap<java.lang.String,java.lang.Double>` | `CorrelationManager.getYValues(ManagerInput<java.lang.String,java.lang.String> input, java.io.PrintWriter out)`             Returns the parsed value for `CorrelationManager.Y_FILE_NAME` in the provided input and prints a descriptive message to the provided print writer. |
| `void` | `MannWhitneyUManager.setInput(ManagerInput<java.lang.String,java.lang.String> input, java.io.PrintWriter out)`             Uses the provided input to prepare this process manager for execution. |
| `void` | `KendallTauManager.setInput(ManagerInput<java.lang.String,java.lang.String> input, java.io.PrintWriter out)`             Uses the provided input to prepare this process manager for execution. |
| `void` | `CorrelationManager.setInput(ManagerInput<java.lang.String,java.lang.String> input, java.io.PrintWriter out)`             Uses the provided input to prepare this process manager for execution. |

Uses of ManagerInput in sbrt.shell.mng.utilities

| Methods in sbrt.shell.mng.utilities with parameters of type ManagerInput | |
| --- | --- |
| `static java.util.List<LinearComb<java.lang.String>>` | `VariableParticipationManager.getExpressions(ManagerInput<java.lang.String,java.lang.String> input, java.io.PrintWriter out)`             Opens the file denoted by `VariableParticipationManager.EXPRESSION_FILE` in the provided manager input and returns the list of mathematical expressions it contains. |
| `void` | `VariableParticipationManager.setInput(ManagerInput<java.lang.String,java.lang.String> input, java.io.PrintWriter out)`             Uses the provided input to prepare this process manager for execution. |
| `void` | `VectorComparisonManager.setInput(ManagerInput<java.lang.String,java.lang.String> input, java.io.PrintWriter out)`             Uses the provided input to prepare this process manager for execution. |
| `void` | `IntervalComparisonManager.setInput(ManagerInput<java.lang.String,java.lang.String> input, java.io.PrintWriter out)`             Uses the provided input to prepare this process manager for execution. |

---


|  |  |  |
| --- | --- | --- |
| **Overview** \| **Package** \| **Class** \| **Use** \| **Tree** \| **Deprecated** \| **Index** \| **Help** | |  |
| PREV   NEXT | **FRAMES**    **NO FRAMES**     **All Classes** |  |


---
